# Artificial intelligence extracts key insights from legal documents to predict intimate partner femicide

**DOI:** 10.1038/s41598-023-45157-5

**Published:** 2023-10-24

**Authors:** Esperanza Garcia-Vergara, Nerea Almeda, Francisco Fernández-Navarro, David Becerra-Alonso

**Affiliations:** 1 Department of Quantitative Methods, Universidad Loyola Andalucía, Sevilla, Spain; 2 Department of Psychology, Universidad Loyola Andalucía, Sevilla, Spain; 3 Department of Computer Languages and Computer Science, University of Málaga, Málaga, Spain

**Keywords:** Human behaviour, Mathematics and computing

## Abstract

Legal documents serve as valuable repositories of information pertaining to crimes, encompassing not only legal aspects but also relevant details about criminal behaviors. To date and the best of our knowledge, no studies in the field examine legal documents for crime understanding using an Artificial Intelligence (AI) approach. The present study aims to fill this research gap by identifying relevant information available in legal documents for crime prediction using Artificial Intelligence (AI). This innovative approach will be applied to the specific crime of Intimate Partner Femicide (IPF). A total of 491 legal documents related to lethal and non-lethal violence by male-to-female intimate partners were extracted from the Vlex legal database. The information included in these documents was analyzed using AI algorithms belonging to Bayesian, functions-based, instance-based, tree-based, and rule-based classifiers. The findings demonstrate that specific information from legal documents, such as past criminal behaviors, imposed sanctions, characteristics of violence severity and frequency, as well as the environment and situation in which this crime occurs, enable the correct detection of more than three-quarters of both lethal and non-lethal violence within male-to-female intimate partner relationships. The obtained knowledge is crucial for professionals who have access to legal documents, as it can help identify high-risk IPF cases and shape strategies for preventing crime. While this study focuses on IPF, this innovative approach has the potential to be extended to other types of crimes, making it applicable and beneficial in a broader context.

## Introduction

In recent decades, the exponential growth of electronic legal information produced in courts has prompted the use of artificial intelligence (AI) to process and manage this vast amount of valuable data. AI aids legal professionals in various tasks such as retrieving, classifying, translating, summarizing, and reviewing legal documents, as well as predicting case outcomes^1, 2^. AI, as a computer science discipline, encompasses the design, development, and implementation of intelligent systems capable of efficiently processing large volumes of data, identifying patterns, solving complex problems, and making informed decisions based on available information^3, 4^. In the legal field, the two main branches of AI, Natural Language Processing (NLP) and Machine Learning (ML), have been extensively applied.

NLP, in particular, involves algorithms to analyze extensive volumes of text-based data, including extracting relevant information as well as interpreting and generating human language^5, 6^. Legal professionals need to read previous similar cases for reference in their own defenses, and NLP assists in this search of legal documents of interest as well as producing summaries to avoid spending long time reading long documents and picking out the useful information^7, 8^. The studies of Hachey and Grover^9^ and Jain et al.^10^ are instances of using NLP for automatically extracting relevant features of legal text judgments for creating summaries of legal documents without human intervention. In the field of legal debates, researchers such as Atkinson, Eliot, and Greenwood, among others^11–15^ have delved into the analysis of optimal argumentation strategies with the use of NLP. This line of research focuses on developing methods capable of exploring the argumentation employed by individuals in criminal proceedings and unraveling the underlying patterns of argumentation in terms of the structure and content of their arguments. This analysis of previous debates and arguments allows us to learn how to make better and more convincing argumentative syntheses.

ML is also a sub-field of AI that focuses on developing algorithms that allow machines to automatically process large data sets and detect patterns or relationships in data to make future predictions or make decisions based on data^16–19^. Most legal studies that apply ML focus on predicting the final outcome of charges and decision in a legal case. Several studies^20–22^, for instance, detect in light of the alleged crime, available evidence, and applicable laws from large sentences the relevant factors related to specific charges (Determining charges involves the process of formally accusing an individual or entity of committing specific offenses or violations).

Another connected area of law research applying ML focuses on the analysis of legal text for forecasting judicial outcomes. For example, the study of Aletras et al.^23^ analyzed textual patterns and features related to legal arguments and facts influencing judicial decisions of the European Court of Human Rights. The obtained models identified relevant patterns leading to specific judicial decisions, and they allowed predicting courts’ decisions with an accuracy of approximately 79%. Similar studies predicting courts’ decisions of Philippine Supreme Court^24^, Brazilian Court^25^, Moroccan Court^26^ among others, have been carried out to improve and streamline the work of legal professionals when raising a defense, filing appeals, and filing lawsuits.

The application of AI in the analysis of legal documents has showcased significant potential in enhancing various aspects of law enforcement and holds promise in the field of criminology as well. Legal documents contain valuable information about criminal offenses, and their analysis can contribute to a better understanding of the dynamics and patterns of specific criminal behaviors. As a matter of fact, some studies have demonstrated the effectiveness of extracting data from legal documents to delve into the characteristics of offenders and their profiles, as well as the risk factors associated with criminal behaviors. For example, the study of Khoshnood et al.^27^ analyzed court documents to identify common characteristics of homicide perpetrators such as mental health, educational level, criminal history, and substance abuse, and, based on them, developed profiles of criminals homicides. Another example is the study of Vatnar et al.^28^ which determined by court judgments a significant association between substance use by offenders and crime perpetration.

To date and to the best of our knowledge, no studies in the field examine legal documents for crime understanding using an Artificial Intelligence (AI) approach. The incorporation of AI in this field of research could aid in automatically identifying relevant characteristics associated with criminal activities, such as criminal records, modus operandi, and locations, thereby assisting in crime prediction. Identifying the factors associated with criminal incidents expands our understanding and serves to anticipate potential crimes in similar conditions. One of the key advantages of AI is its capacity for rigorous and efficient handling of large-scale data. It excels in identifying complex and non-linear patterns within data, making it exceptionally well-suited for thoroughly analyzing extensive legal documents. Hence, the current study aims to analyze pertinent information available in court judgments using NLP and ML methods to predict crime. The specific focus will be on the crime of intimate partner femicide (IPF) due to its prevalence and severe consequences. The findings from this study will be useful for judges, lawyers, police, and victim support services who can utilize the analyzed information to identify high-risk IPF cases and shape effective prevention strategies.

## Data and method

### Database of the study

#### Sample

The sample of this study consists of cases of lethal violence against women by their current or former male intimate partners (referred as IPF and LV) and cases of non-lethal violence against women by their current or former male intimate partners (labelled as N-LV) determined by court judgements. The interest of the study focuses on the detection of factors associated with LV present in court judgements, and it requires the inclusion of N-LV cases for comparison and validation purposes. The study will also determine which factors are exclusive to LV cases. This comparative analysis will contribute to a better understanding of the differentiated factors of both groups that assist in the detection of IPF.

The cases were extracted from the Vlex legal database by conducting an extensive search of legal documents within this particular database using NLP. The search of cases was conducted on February 13th 2022 and updated on January 3rd 2023. Search criteria includes crime, judicial resolution, jurisdiction, jurisdictional organ, time, and location. By applying NLP algorithms, we were able to perform a sophisticated and context-aware search that filtered through a vast amount of legal documents, pinpointing cases that were directly relevant to our research objectives.

According to the “crime” search criteria, the keyword “gender violence” was introduced in the the database. The inclusion of this term was made by the fact that, within the Vlex database, this term encompasses both LV and N-LV. To be more precise, the specific offenses of the Spanish penal code with their respective penal articles applicable to the crime of LV and N-LV were also incorporated: homicide and murder (articles 138, 139, and 140), injuries (articles 147, 148, 149, 150, and 153), illegal detention (articles 163, 164, and 166), threats (article 169), constrains (article 172), habitual violence (article 173), sexual aggression and abuse (articles 178, 179, 180, 181), prostitution and sexual exploitation (article 187), unauthorized access to privacy (article 197), and insults (articles 208 and 209), which are applicable to LV and N-LV. Consequently, we exclude all crimes not related to LV or N-LV.

With regards to “judicial resolution” search criteria, only final court judgments were selected, whereas provisional resolutions were excluded. The “jurisdiction” search criteria was limited to penal field, while civil, fiscal, social, constitutional, commercial and business, public and administrative, and procedural law fields were excluded.

Concerning “jurisdictional organ” search criteria, only provincial and supreme courts were considered given their competence in various types and severity of LV and N-LV cases. In Spain, instruction courts and specialized courts of violence against women are competent for diligence labor and prosecution of minor offences. Penal courts prosecute crimes with prison punishment of up to 5 years, and thus are excluded due to the medium to severe crimes of N-LV, and LV (that exceed the 5 year limit). Supreme courts can prosecute any LV and N-LV case appealed from lower courts, providing an updated sentence. They mostly have the final decision in these cases. The inclusion of this court increases the diversity of types and severity of LV and N-LV crimes. Moreover, the provincial courts were also included for the same reason: being competent for prosecuting crimes with prison punishment above five years and other N-LV cases that have been appealed, some of them not going to the high courts, such as the supreme court.

Regarding “location” as a search criterion, all the included cases were restricted to Spain as its legal resolutions are differentiated from those in other countries, for both LV and N-LV. Finally, the “time” search criterion concerns with cases from 2019 to 2022 in order to obtain pre and post COVID pandemic data.

After conducting the search strategy, 8481 cases of LV and N-LV were obtained. We read the proven facts and judgement from each sentence. Exclusion criteria were: (i) acquittal sentences (In this study, acquittal sentences are considered to be those in which there is no conviction for LV or N-LV because there is no evidence to prove the occurrence of such crimes. These sentences are not of interest in this study since when the commission of LV or N-LV crimes is not established, they do not provide relevant information on this criminal phenomenon under study), (ii) condemnatory sentences involving adult aggressors and minor victims, (iii) condemnatory sentences for cases of reciprocal violence among men and woman, (iv) sentences that nullify previous judicial decisions of recourse and ordering the continuation of proceedings by the competent judicial organ, (v) sentences that do not provide proven facts to refer the previous sentence of judicial recourse and (vi) sentences written in co-official languages of Spain.

Consequently, a total of 491 cases met the inclusion criteria of LV and N-LV established by penal court judgements and declared by the Spanish provincial and supreme courts. Out of the 491 cases, 330 are N-LV and 161 are LV. Accordingly, the 330 cases of N-LV involve crimes against personal freedom, physical and psychological integrity, sexual integrity, and privacy. The remaining 161 cases are related to homicides and murders. All of them perpetrated against women by men who are their husbands, ex-husbands, partners or an ex-partners.

Regarding the sampling strategy, for those mentioned above, a stratified random sampling approach was adopted to ensure the dataset’s representativeness. The process randomly selects sentences from the LV and N-LV categories while preserving the original class proportions found in the population. This approach allowed us to maintain a balanced dataset and prevent biases arising from an imbalanced class distribution.

#### Variables

The variables and associated data have been gathered from the 491 penal court judgments declared by the Spanish provincial and supreme courts, as detailed in the preceding “Sample”. Consequently, the reliability of the information in the study depends on the reliability of the data extracted from these court judgments.

The dependent variable is IPF which is categorized into two values: 0 and 1. A value of 0 means absence (In this study, the absence of a specific variable is not the same as missing data. When a court judgment does not provide information about a specific variable, that is, it is considered a missing value when the variable is not mentioned in such a legal document. In contrast, we considered absence when the legal document refers to the variable but indicates that it is not fulfilled or present in the situation described) of LV (or presence of N-LV), whereas a value 1 signifies presence of LV. A total of 33 independent variables were included in this study, which have been organized into 3 groups. The first group includes variables related to past criminal behaviours and sanctions imposed for their commission. The second group involves variables referred to environment and situation where the LV and N-LV crimes occur. The third group consists of variables related to characteristics of the violence against women perpetrated by their intimate partners.

The first group consists of 23 independent variables. On the one hand, there are 11 variables (number 1–11) related to past criminal history. These variables correspond to past criminal behaviours proven and convicted by court judgements. Variable number 1 contains criminal history not related to intimate partner violence against women by males. Variable number 2 involves exclusively prior criminal behaviours related to this intimate partner violence. Variables number 3–11 detail which specific crimes against women by male intimate partners were perpetrated (Variable number 2 was introduced in the study to examine the connection between previous criminal behaviors related to intimate partner violence and the perpetration of IPF. Additionally, variables from number 3 to 11 were included in a more detailed exploration of this issue, aiming to analyze whether specific aspects of a criminal history related to intimate partner violence exhibit a stronger association with IPF). The remaining 12 variables are related to sanctions imposed for the perpetration of these criminal behaviours towards women in the relationship. The variables 12–17 specify the types of sanctions imposed, and variables 18–23 the length of said sanctions. The literature revealed that prior criminal behavior is a strong and reliable predictor of future criminal behavior^29, 30^. Therefore, examining past criminal behaviors in the study of LV and N-LV is essential. Previous studies have delved into this issue and indicate that criminal records are associated with LV^31–33^. However, to our knowledge, it has not been analyzed which specific types of past criminal behaviors are related to LV. Furthermore, no evidence of the impact of previously imposed sanctions on the commission of LV and N-LV has been analyzed. For this reason, the current study analyses the variables of past criminal behaviors and sanctions imposed for their commission.

1. General criminal history (value 0 for absence and value 1 for presence).
2. Specific criminal history (value 0 for absence and value 1 for presence).
3. Criminal history of injuries (value 0 for absence and value 1 for presence) (According to the Spanish penal code, injury crimes consist of “causing, by any means or procedure, damage that impairs bodily integrity or physical or mental health”).
4. Criminal history of threats (value 0 for absence and value 1 for presence) (According to the Spanish penal code, threat crimes imply “announcing to someone the intention of causing him/her, his/her family or other persons of his/her closest relationships a harm that constitutes crimes of homicide, injury, abortion, against freedom, torture and against moral integrity, sexual freedom, intimacy, honor, patrimony and socioeconomic order”).
5. Criminal history of constraints (value 0 for absence and value 1 for presence) (According to the Spanish penal code, constraining crime consists of “using violence, without being legitimately authorized, to prevent another person from doing something that the law does not prohibit, or to compel him/her to do something that this person does not want to do, whether just or not”).
6. Criminal history of habitual violence (value 0 for absence and value 1 for presence) (According to the penal code, habitual violence crimes involve inflicting on another person degrading treatment, seriously undermining his/her moral integrity repeatedly over time (a specific number is not specified)).
7. Criminal history of insults (value 0 for absence and value 1 for presence) (According to the Spanish penal code, insult crimes concerns the action or expression (oral or written) that seriously injures a person’s dignity).
8. Criminal history of illegal detention (value 0 for absence and value 1 for presence) (According to the Spanish penal code, illegal detention crime includes “locking up or detaining another person, depriving her of her liberty”).
9. Criminal history of sexual aggression (value 0 for absence and value 1 for presence) (According to the Spanish penal code, sexual aggression crimes refer to attacks against sexual freedom of a person through the use of violence or intimidation).
10. Criminal history of sexual abuse (value 0 for absence and value 1 for presence) (According to the Spanish penal code, sexual abuse crimes are the same as sexual aggression but do not imply violence or intimidation).
11. Criminal history of invasion of privacy (value 0 for absence and value 1 for presence) (According to the Spanish penal code, crimes against personal privacy entail the unauthorized access and control of personal information).
12. Prison sentence (value 0 for absence and value 1 for presence).
13. Deprivation of the right to possess and carry weapons sentence (value 0 for absence and value 1 for presence).
14. Prohibition to approximate the victim sentence (value 0 for absence and value 1 for presence).
15. Prohibition to communicate with the victim sentence (value 0 for absence and value 1 for presence).
16. Community service sentence (value 0 for absence and value 1 for presence).
17. Fine sentence (value 0 for absence and value 1 for presence).
18. Time of prison (in years).
19. Time of deprivation of the right to possess and carry weapons (in years).
20. Time of prohibition to approximate the victim sentence (in years).
21. Time of prohibition to communicate with the victim sentence (in years).
22. Time of community service sentence (in days).
23. Time of fine sentence (in days).

The second group consists of 5 independent variables that capture the environment and situational circumstances of LV and N-LV crimes (number 24–28). Although past research underscores the significance of considering the environment and its contextual elements in the study of criminal behavior^34, 35^, a limited number of studies have explored this association with LV. The existing ones have exclusively focused on geographic context and characteristics of the neighborhood where LV occurs^36–40^. The current study strives to broaden the scope by including additional environmental and situational variables, thus contributing to a better understanding of the environment and situation with regard to LV.

24. Place of crime indicates whether the commission of LV or N-LV crime occurred in a rural (value 0) or urban area (value 1).
25. Presence of people concerns whether the LV or N-LV crime occurs in an area where commonly there is nobody or few people around (e.g., a dead-end street) (value 0), or on the other hand, if there are more people present (e.g., a main avenue) (value 1).
26. Social control refers to the presence of people who are aware of the occurrence of a LV or N-LV crime while it is being committed, and have the influence to discourage criminal behavior. It involves formal control including professionals (e.g., police) and informal control referring to non-professionals (e.g., family members and neighbors) (value 0 for absence of social control and value 1 for its presence).
27. Time of crime refers to whether the LV or N-LV crime was committed in dawn (value 1), morning (value 2), afternoon (value 3), night (value 4), or at various moments (value 5).
28. Dispute refers to conflicts between offender and victim during the commission of the LV or N-LV crime or just before the crime began (value 0 for absence of dispute and value 1 for presence of it).

The third group involves 5 independent variables that capture the characteristics of the violence against women perpetrated by their intimate partners (number 29 to 33). Prior research has delved into various variables encompassing the frequency and severity of violence, the intensification of violent incidents, and the types of violence associated with LV. These studies consistently highlight the significant association between these factors and LV, underscoring their relevance to the current study^41–43^. By contrast, concerning coping strategies, previous studies have explored their impact on N-LV. Their findings suggest that disengagement coping strategies are related to elevated risk and prevalence of N-LV^44, 45^. However, to the best of our knowledge, there is a lack of research analyzing the influence of coping strategies on LV. This critical gap in the literature has driven our decision to incorporate this variable into our present study.

29. Frequency of violence involves the number of violent incidents occurs. It have been categorized into three levels: low (1 violent incident) (value 1), medium (2–3 violent incidents) (value 2), and high (4 times and above) (value 3).
30. Escalation of violence refers to the gradual rise in frequency and severity of violence as time goes on. The temporal sequence of the criminal behaviors related to intimate partner violence was analyzed, and in the presence of a crime considered by the Spanish code as a minor crime at time point 1 and a severe crime at time point 2, the variable takes value 1. Otherwise, the variable takes a value of 0.
31. Physical violence refers to assaults that inflict harm or pose a threat to victims’ physical integrity such as punching, strangling, and shooting (value 0 for absence and value 1 for presence).
32. Psychological violence involves diverse actions that impact on victims’ mental health such as acts and expressions that inflict humiliation, fear, and undermine the value and dignity (value 0 for absence and value 1 for presence).
33. Victim’s coping strategies in the face of violence refers to the actions taken by the victim to deal with the violence: disengagement coping strategies (value 0) and engagement coping strategies (value 1). Disengagement coping strategies are assigned when the victim takes little or no action to deal with the violence, such as submission attitudes and conflict avoidance. On the other hand, engagement coping strategies refers to when the victim deals with violence by using active behaviors such as seeking for information and help, denouncing, and leaving the relationship.

Once the variables were collected and prior to develop the analysis, data cleaning process was carried out to ensure that numerical variables were within the range established by the researchers. It is important to note that at the beginning of the study, 49 independent variables were considered, but 16 variables with a significant number of missing values (age, birthdate, marital status, employment status, and mental disorders of both offenders and victims; permanent location sentence, disqualification sentence, deprivation of the right to drive motor vehicles and motorcycles sentence, deprivation of the right to reside in or go to concrete places, instruments of crime, and breaking sentence(s)). Therefore, these 16 variables were excluded from the analysis and not explained in this section. Thus, only the dependent variable and 33 independent variables included in this section were considered in the study.

### Data analysis

#### Algorithms used in the study

In this experimental study, we have explored the performance of 14 classifiers belonging to diverse families of models. These classifiers were implemented in Weka, a popular machine learning toolkit. The models have been defined based on the distinct families considered, encompassing Bayesian classifiers, function-based classifiers, instance-based classifiers, tree-based classifiers, and rule-based classifiers^46^.

Bayesian classifiers (2 classifiers). Bayesian classifiers are a family of classification algorithms based on the application of Bayes’ theorem. They calculate the probability of an instance belonging to a particular class by considering the probabilities of its features given each class. In this study, we have implemented two examples of Bayesian classifiers to explore their effectiveness in classifying the real-world problem of the research study:

1. *BayesNet*. BayesNet is a classification algorithm that implements a Bayesian network model. It uses probabilistic dependencies between features to make predictions. It is based on the Bayes’ theorem and employs a network structure to represent conditional dependencies among variables, allowing for efficient and accurate classification tasks.
2. *NaivesBayes*. NaiveBayes is another classification algorithm that is based on the Bayes’ theorem. It assumes that all features are independent of each other given the class variable. This “naive” assumption simplifies the computation, making it computationally efficient. Unlike BayesNet, NaiveBayes does not consider the dependencies between features, which can lead to less accurate predictions in scenarios where feature interdependencies exist.

Functions-based classifiers (5 classifiers). Function-based classifiers, such as logistic regression, neural networks, and support vector machines (SVM), are powerful algorithms that learn a mathematical function to map input features to class labels. In this study, we have considered five examples of function-based classifiers:

3. *Logistic regression*. The Logistic regression classifier is a popular algorithm used for binary classification tasks. It is based on the logistic regression model and uses a sigmoid function to estimate the probability of an instance belonging to a particular class. It fits a linear decision boundary by maximizing the likelihood function, making it suitable for problems where the relationship between features and the target variable is assumed to be linear.
4. *SimpleLogistic*. The SimpleLogistic classifier is an extension of the Logistic classifier. It combines logistic regression with feature selection by using a backward elimination process. It starts with a full set of features and removes them one by one based on their contribution to the model’s performance, resulting in a simpler and potentially more interpretable model. SimpleLogistic can be useful when dealing with datasets with a large number of features and aiming to find a compact yet accurate model.
5. *MultilayerPerceptron*. The MultilayerPerceptron classifier is a versatile and powerful algorithm for both classification and regression tasks. It is based on artificial neural networks and uses multiple layers of interconnected nodes (neurons) to learn complex relationships between features and the target variable. With adjustable hidden layers, activation functions, and learning parameters, it can effectively capture non-linear patterns in data and adapt to different problem domains. It is especially useful when dealing with large-scale datasets and complex decision boundaries.
6. *RBFClassifier*. The RBFClassifier is a classifier based on radial basis function (RBF) networks. It uses a set of radial basis functions to transform the input data into a higher-dimensional space. Each function represents a prototype or center point, and the classifier assigns instances to the class associated with the nearest prototype. RBFClassifier is effective for nonlinear classification tasks and can handle complex decision boundaries, making it suitable for datasets with intricate relationships between features and the target variable.
7. *SMO*. The SMO (Sequential Minimal Optimization) classifier is a fast and efficient algorithm for training support vector machines (SVMs). It solves the quadratic optimization problem by decomposing it into a series of smaller sub-problems. SMO is known for its ability to handle large datasets and high-dimensional feature spaces effectively. It is a popular choice for binary classification tasks and can handle both linearly separable and non-linearly separable data using different kernel functions.

Instance-based classifiers (2 classifiers). Instance-based classifiers are algorithms that make predictions based on the similarity between instances in the training data and the test instance. In the study, we implemented two classifiers from this family of models:

8. *IBk*. The Ibk classifier is an instance-based learning algorithm that utilizes the k-nearest neighbors (k-NN) approach for classification. It assigns a class label to an instance based on the majority vote of its k nearest neighbors in the training set. Ibk is a versatile classifier that can handle both numerical and categorical attributes. It is particularly useful for datasets with local patterns or where instances with similar attribute values tend to belong to the same class.
9. *KStar*. Kstar is a classifier that belongs to the family of instance-based learning algorithms. The classification of a test instance is determined by examining the class labels of similar training instances, as measured by a similarity function. What sets Kstar apart from other instance-based learners is its utilization of an entropy-based distance function for calculating the similarity between instances.

Tree-based classifiers (4 classifiers). Tree-based classifiers are a group of algorithms that construct decision trees to make predictions. These classifiers partition the feature space into smaller regions based on feature values and use a tree structure to navigate through the decision-making process. In the study, we have evaluated the performance of four classifiers from this family of models:

10. *LMT*. The LMT (Logistic Model Trees) classifier is a hybrid model that combines decision trees with logistic regression. It constructs a decision tree where each leaf node contains a logistic regression model. This approach allows LMT to capture both linear and non-linear relationships between features and the target variable. LMT is particularly useful when dealing with datasets that have complex interactions among variables, offering a balance between interpretability and predictive accuracy.
11. *RandomTree*. The RandomTree classifier is designed to construct a tree by considering a subset of K randomly chosen attributes at each node. It does not perform any pruning techniques. Additionally, it offers the option to estimate class probabilities (or target mean for regression) using a hold-out set, a process known as backfitting. This feature allows for improved estimation of class probabilities or target values during the training process.
12. *RandomForest*. The RandomForest classifier is an ensemble learning algorithm that combines multiple decision trees to make predictions. Each tree is constructed using a random subset of features and a bootstrap sample of the training data. The final prediction is determined by aggregating the predictions of all trees, resulting in improved accuracy and robustness against overfitting. RandomForest is widely used for classification and regression tasks, particularly for handling complex datasets with high-dimensional feature spaces.
13. *J48*. The J48 classifier is an implementation of the C4.5 algorithm, which constructs decision trees based on information gain. It recursively splits the data based on the best attribute at each node, aiming to maximize the separation of classes. J48 handles both categorical and numerical attributes and supports pruning to avoid overfitting. It is a popular and widely used classifier due to its simplicity, interpretability, and ability to handle both binary and multi-class classification problems.

Rule-based classifier (1 classifier). Rule-based classifiers are a category of algorithms that generate if-then rules to make predictions. These classifiers use a set of rules that describe the relationships between features and class labels. Within this group of models, we have focused our attention on a single model:

14. *JRip*. The JRip classifier is a rule-based classifier that generates a set of if-then rules to make predictions. It uses a modified version of the RIPPER algorithm and applies a two-phase optimization process to construct accurate and compact rule sets. JRip is particularly useful when interpretability is important, as the generated rules can provide insights into the decision-making process. It is suitable for both binary and multi-class classification tasks and performs well on datasets with discrete or categorical attributes.

In the implementation of the algorithms, cost-sensitive learning was utilized to incorporate the cost of misclassifications. A weight of 2 was assigned to false positives (cases of LV incorrectly classified as N-LV), while a weight of 1 was assigned to false negatives (cases of N-LV incorrectly classified as LV). This weighting scheme ensured that the penalty for false positives was more severe than for false negatives. This approach was necessary due to the inherent class imbalance between LV and N-LV classes. Without employing this process, the algorithms tended to prioritize the recognition of the majority class (N-LV class). However, it is crucial to accurately identify the minority class as well, achieving a balanced classification of both classes.

To ensure that only informative variables were included in the analysis, and not redundant factors, a straightforward feature selection algorithm was applied, Recursive Feature Elimination (RFE)^47^. RFE iteratively removes the least significant features, ranking them based on their contribution to the model’s performance. This technique helped us focus on the most relevant variables and avoid redundancy in our analysis, resulting in a more streamlined and interpretable model.

#### Experimental design

To ensure the reliability of the outcomes, a hold-out as a specific form of resampling technique was applied to the final sample (491 cases), dividing the data into two sets. The first set, which included 66% of the total sample (324 cases), was used for a training classification model. The second set consisted of 34% of the sample and was used for the model testing (167 cases). This splitting was conducted 30 times, randomizing by altering the seed of the set partitioner.

As for the specific split of 66% for training and 34% for testing, this choice was made based on a combination of prior research findings and best practices in machine learning. Numerous studies, such as Fernández-Navarro et al. and Gutiérrez et al.^48, 49^, have shown that a 66–34% split strikes a reasonable balance between model training and testing while avoiding overfitting. This choice is consistent with industry standards and helps ensure the model’s performance generalizes well to unseen data. The decision to repeat the experiment 30 times, rather than 50 or 100, was influenced by practical considerations and computational resources. While a larger number of repetitions can provide more stable results, 30 repetitions have been found to yield reliable outcomes, as demonstrated in studies like Perales-González et al. and Durán-Rosal et al.^50, 51^. Additionally, this number allowed us to balance computational efficiency and robustness in the analysis.

An incremental approach is adopted in analyzing the independent variables to comprehensively examine the LV and N-LV, considering their multifaceted nature. The study examines whether considering more variables can help differentiate between LV and N-LV. Accordingly, the study examines whether the detection of LV and N-LV is more accurate when a broad set of variables is considered. This incremental progression is based on the need to unravel the complex factors associated with LV and N-LV and ultimately obtain a more holistic understanding of the phenomenon under study. Thus, all the aforementioned algorithms were utilized in three distinct phases, with each phase repeated 30 times using the hold-out process. During Phase 1 of the study, the algorithms were applied solely to the independent variables of Group 1. Phase 2 extended the analysis to include the independent variables from both Group 1 and Group 2. Lastly, in Phase 3, the algorithms incorporated the independent variables from Group 1, Group 2, and Group 3, as outlined in detail in “Variables”.

#### Measures

All the metrics considered in our evaluation are derived from the confusion matrix. The reason for this choice is the diversity of families of classifiers included in our study. While these classifiers may have varying output formats, they all provide information about the predicted category for each instance. By utilizing this information, we can construct the confusion matrix, which enables us to calculate metrics such as accuracy, precision, recall, and F-score^52^. This approach ensures a consistent and comprehensive evaluation across all classifiers, facilitating meaningful comparisons and providing valuable insights into their performance.

A confusion matrix affords a comprehensive overview of a classification model’s performance. This matrix illustrates in a table the connection between predicted and actual classifications within a specific data set, allowing an evaluation of the precision of the model and the errors it produce. The confusion matrix used in the study is shown in Table 1 which involves the following elements: (i) number of true positives (Number of instances of N-LV classified correctly, $n_{11}$), (ii) number of true negatives (number of instances of LV classified correctly, $n_{22}$) (iii) Number of false positives (number of instances of LV classified incorrectly as N-LV class, $n_{21}$) and (iv) Number of false negatives (number of instances of N-LV classified incorrectly as LV class, $n_{12}$).

**Table 1:** Confusion matrix of the LV and N-LV classification problem.

		Estimated classification	
		Non-lethal violence	Lethal violence
Actual classification	Non-lethal violence	$n_{11}$	$n_{12}$
	Lethal violence	$n_{21}$	$n_{22}$

The specific metrics derived from the confusion matrix considered in the study are the following (see^52^ for more detailled information):

$$S_{1}$$
Correct classification for N-LV class or class 1. It is also called as the sensitivity of the first class in^52^. Mathematically, it is defined as:
  $$\begin{aligned} S_1 = \frac{n_{11}}{n_1}, \end{aligned}$$
  where $n_1$ is the total number of patterns belonging to class 1.
$$S_{2}$$
Sensitivity for LV class or class 2^52^. In this case, the percentage of correctly classified LV patterns, which is defined as:
  $$\begin{aligned} S_2 = \frac{n_{22}}{n_2}, \end{aligned}$$
  where $n_2$ is the total number of patterns belonging to LV class.
CCR: The correct classification rate (CCR) often referred to as accuracy, is a metric used in classification tasks to measure the proportion of correctly classified instances out of the total number of instances in a dataset. It is a fundamental performance measure in machine learning and is typically represented as a percentage. Formally, the correct classification rate (CCR) can be defined as:
  $$\begin{aligned} CCR = \frac{n_{11} + n_{22}}{n_1 + n_2}. \end{aligned}$$
F-score: The F-score, also known as the $F_1$-score, is a commonly used metric in binary (two-class) classification problems. It combines precision and recall to provide a single value that balances the trade-off between these two metrics. Mathematically, the F1-score is defined as:
  $$\begin{aligned} F_{1} = \frac{2\cdot P \cdot R}{P+R} \end{aligned}$$
  where *P* is the precision, defined as:
  $$\begin{aligned} P = \frac{n_{11}}{n_{11}+ n_{21}} \end{aligned}$$
  and *R* is the recall, defined as:
  $$\begin{aligned} R=\frac{n_{11}}{n_{11} + n_{12}} \end{aligned}$$
AA: The Average Accuracy (AA) is the arithmetic average per-class effectiveness of a classifier. It is usually referred as macro-average^53^. The AA (macro average) is a metric commonly used in imbalanced classification scenarios. In imbalanced datasets, where one class significantly outnumbers the others, accuracy alone may not provide an accurate representation of a model’s performance. In such cases, macro-averaging the accuracy can offer valuable insights, since by using macro-averaged accuracy, you give equal importance to each class, regardless of its size. Mathematically, it is defined as:
  $$\begin{aligned} AA = \frac{S_1 + S_2}{2} \end{aligned}$$
GM: The Geometric Mean corresponds to the geometric average of the partial accuracies for each class. GM is another metric applicable in imbalanced classification scenarios, and it exhibits greater sensitivity to unbalanced classifications within the baseline classes (N-LV and LV) when compared to macro-average accuracy (AA). GM is mathematically defined as follows:
  $$\begin{aligned} GM = \sqrt{S_1 \cdot S_2} \end{aligned}$$

## Results

The three analyses presented in this study yielded results that are summarized in Tables 2, 3, and 4. Table 2 presents the outcomes of analysis 1, focusing on the classification of lethal and non-lethal violence against women by their partners using only variables related to past criminal behaviors and imposed sanctions. Analysis 2 results are presented in Table 3, and incorporates additional variables related to the environmental and situational context of the crimes alongside the variables from analysis 1. Table 4 showcases the findings of analysis 3, combining variables from both analyses 1 and 2, along with supplementary variables pertaining to the characteristics of violence against women perpetrated by their intimate partners. Each table presents the mean and standard deviation results of the 30 runs of each algorithm on the respective test sets for each analysis conducted in the study. The best scores per metric are highlighted in bold, while the second-best scores are indicated in italics.

In general, the results for $S_{1}$, $S_{2}$, CCR, F-score, AA, and GM metrics in Table 3 exhibit better performance compared to those in Table 2. Furthermore, the results in Table 4 surpass those in both Tables 2 and 3. This improvement signifies that incorporating a larger number of variables leads to an enhanced detection of both lethal and non-lethal cases. In other words, the inclusion of additional variables in the analysis contributes to a more accurate classification process, resulting in better identification of both types of cases.

Regarding the $S_{1}$ metric, Table 2 demonstrates that the IBk algorithm achieves the highest performance in detecting the non-lethal class with a detection rate of 54.27%. However, in both Tables 3 and 4, the RandomForest algorithm outperforms the others in detecting the non-lethal class. In Table 3, the detection rate for this class reaches 74.16%, while it further increases to 87.04% in Table 4. These results indicate that the RandomForest algorithm consistently demonstrates improved performance in accurately identifying non-lethal cases across the analyses, with a higher detection rate in Table 4 compared to Table 3, where more variables are considered.

In terms of the $S_{2}$ metric, Table 2 reveals that the RandomForest algorithm outperforms the others in accurately identifying the lethal class, detecting 68.49% of lethal cases. However, both Tables 3 and 4 indicate that the BayesNet algorithm exhibits the highest effectiveness in detecting this class. In Table 3, BayesNet achieves a detection rate of 70.56% for the lethal class, and in Table 4, this rate further improves to 82.36%, underscoring the consistently superior performance of BayesNet in detecting the lethal class.

Considering the CCR metric, Table 2 demonstrates that the BayesNet algorithm achieves the best performance in accurately classifying both the lethal and non-lethal classes, with a CCR score of 59.26%. In Table 3, the RBFClassifier algorithm correctly classifies 71.10% of both lethal and non-lethal cases, indicating an improvement in performance compared to Table 2. However, in Table 4, the RandomForest algorithm achieves the highest CCR score of 83.16%, representing a significant improvement in performance compared to both Tables 2 and 3.

In terms of the F-score, the RBFClassifier algorithm demonstrates notable performance in both Tables 2 and 3, exhibiting improved precision and recall compared to other algorithms. In Table 2, the RBFClassifier achieves an F-score of 63.05, while in Table 3, it notably improves to 77.23. However, in Table 4, the RandomForest algorithm outperforms other algorithms with an F-score of 87.40. This signifies the high effectiveness of RandomForest in accurately identifying classes and capturing the majority of cases per class.

The AA scores obtained for each algorithm demonstrate an overall improvement in the identification of both classes throughout the study analyses. In Table 2, the RBFClassifier algorithm achieves the highest AA score of 60.87, indicating a moderate level of accuracy in correctly detecting instances across the lethal and non-lethal classes. Similarly, in Table 3, the RBFClassifier maintains its superior performance with an AA score of 70.03, representing a notable improvement compared to Table 2. However, in Table 4, the RandomForest algorithm surpasses the others, exhibiting the highest AA score of 81.14%. This significant improvement in overall accuracy emphasizes the algorithm’s capability to accurately detect both classes. Furthermore, the tables demonstrate an increasing trend in GM scores, with the RBFClassifier algorithm consistently achieving the highest scores across all study analyses. Specifically, in Table 2, the RBFClassifier attains a score of 25.84. This score improves to 34.61 in Table 3 and further increases to 46.44 in Table 4.

**Table 2:** Mean and standard deviation of $S_{1}$, $S_{2}$, CCR, AA, GM and F-score metrics considering the variables of past criminal behaviors and sanctions.

	$S_{1}$	$S_{2}$	CCR	F-score	AA	GM
BayesNet	$60.76_{20.52}$	$56.24_{26.00}$	$\mathbf {59.26}_{\mathbf {5.78}}$	$64.93_{10.49}$	$58.50_{23.26}$	$24.16_{3.77}$
NaivesBayes	$65.04_{31.45}$	$44.00_{33.28}$	$58.13_{10.75}$	$62.71_{21.29}$	$54.52_{32.11}$	$20.23_{7.29}$
Logistic	$49.37_{15.19}$	$67.19_{18.69}$	$55.20_{4.85}$	$58.54_{8.67}$	$58.28_{16.94}$	$23.46_{2.01}$
SimpleLogistic	$53.63_{23.38}$	$62.06_{25.42}$	$56.38_{7.71}$	$59.79_{12.80}$	$57.82_{24.40}$	$23.52_{4.20}$
MultilayerPerceptron	$49.48_{13.48}$	$68.86_{13.99}$	$55.84_{5.13}$	$59.20_{7.87}$	$59.17_{13.74}$	$24.09_{1.33}$
RBFClassifier	$\textit{53.73}_{\textit{9.40}}$	$68.01_{10.32}$	$\textit{58.42}_{\textit{3.90}}$	$\mathbf {63.05}_{\mathbf {5.70}}$	$\mathbf {60.87}_{\mathbf {9.86}}$	$\mathbf {25.84}_{\mathbf {0.07}}$
SMO	$59.40_{24.78}$	$53.39_{26.02}$	$57.43_{8.50}$	$62.55_{13.67}$	$56.39_{25.40}$	$22.42_{0.4.56}$
IBk	$\mathbf {54.27}_{\mathbf {4.13}}$	$61.87_{7.24}$	$56.76_{2.85}$	$\textit{62.70}_{\textit{3.13}}$	$58.07_{5.68}$	$23.74_{0.02}$
KStar	$45.26_{14.06}$	$69.00_{16.50}$	$53.07_{5.30}$	$55.34_{9.21}$	$57.13_{15.28}$	$22.08_{1.64}$
LMT	$49.52_{16.42}$	$66.75_{18.26}$	$55.19_{5.86}$	$58.43_{9.59}$	$58.14_{17.34}$	$23.37_{2.12}$
RandomTree	$49.72_{5.16}$	$\textit{67.21}_{\textit{6.93}}$	$55.46_{3.03}$	$59.85_{4.02}$	$58.47_{6.05}$	$23.63_{0.03}$
RandomForest	$50.64_{6.53}$	$\mathbf {68.49}_{\mathbf {6.82}}$	$56.50_{3.48}$	$60.76_{5.02}$	$\textit{59.57}_{\textit{6.68}}$	$\textit{24.53}_{\textit{0.03}}$
J48	$49.31_{14.69}$	$65.17_{15.78}$	$54.50_{5.38}$	$58.25_{8.65}$	$57.24_{15.24}$	$22.72_{1.64}$
JRip	$50.73_{11.05}$	$61.67_{13.50}$	$54.32_{4.14}$	$59.27_{6.71}$	$56.20_{12.28}$	$22.12_{1.06}$

Significant values are in bold and italics.

**Table 3:** Mean and standard deviation of $S_{1}$, $S_{2}$, CCR, AA, GM and F-score metrics considering the variables of past criminal behaviors, sanctions, and environment and situation of crime.

	$S_{1}$	$S_{2}$	CCR	F-score	AA	GM
BayesNet	$67.95_{5.31}$	$\mathbf {70.56}_{\mathbf {7.79}}$	$68.80_{2.44}$	$74.44_{2.68}$	$\textit{69.25}_{\textit{6.55}}$	$33.90_{0.03}$
NaivesBayes	$66.95_{19.10}$	$59.55_{23.65}$	$64.53_{7.31}$	$70.19_{11.50}$	$63.25_{21.37}$	$28.19_{3.19}$
Logistic	$71.81_{5.28}$	$65.33_{7.61}$	$69.68_{3.12}$	$76.01_{3.06}$	$68.57_{6.44}$	$33.18_{0.03}$
SimpleLogistic	$72.03_{9.44}$	$66.04_{9.36}$	$\textit{70.07}_{\textit{4.27}}$	$76.05_{5.06}$	$69.04_{9.40}$	$33.64_{0.06}$
MultilayerPerceptron	$\textit{74.19}_{\textit{4.50}}$	$58.83_{8.21}$	$69.14_{3.17}$	$\textit{76.31}_{\textit{2.74}}$	$66.51_{6.36}$	$30.86_{0.03}$
RBFClassifier	$73.15_{4.46}$	$66.91_{7.83}$	$\mathbf {71.10}_{\mathbf {2.74}}$	$\mathbf {77.23}_{\mathbf {2.48}}$	$\mathbf {70.03}_{\mathbf {6.15}}$	$\mathbf {34.61}_{\mathbf {0.02}}$
SMO	$71.19_{8.07}$	$67.10_{9.92}$	$69.84_{3.16}$	$75.80_{3.84}$	$69.14_{8.99}$	$\textit{33.78}_{\textit{0.06}}$
IBk	$65.21_{3.59}$	$65.02_{6.81}$	$65.15_{3.28}$	$71.51_{2.84}$	$65.12_{5.20}$	$29.98_{0.02}$
KStar	$63.75_{5.27}$	$61.80_{8.09}$	$63.11_{3.09}$	$69.80_{3.24}$	$62.78_{6.68}$	$27.86_{0.03}$
LMT	$66.84_{5.81}$	$60.03_{7.34}$	$64.61_{4.02}$	$71.62_{3.91}$	$63.44_{6.57}$	$28.37_{0.03}$
RandomTree	$71.10_{5.15}$	$55.06_{6.36}$	$65.83_{3.32}$	$73.57_{3.11}$	$63.08_{5.75}$	$27.68_{0.02}$
RandomForest	$\mathbf {74.16}_{\mathbf {3.35}}$	$55.90_{7.56}$	$68.17_{2.59}$	$75.77_{2.04}$	$65.03_{5.45}$	$29.32_{0.02}$
J48	$60.61_{6.74}$	$\textit{67.77}_{\textit{8.92}}$	$62.95_{2.76}$	$68.53_{3.79}$	$64.19_{7.83}$	$29.04_{0.04}$
JRip	$67.59_{6.01}$	$68.84_{11.78}$	$68.00_{3.44}$	$73.85_{3.27}$	$68.22_{8.89}$	$32.90_{0.05}$

Significant values are in bold and italics.

**Table 4:** Mean and standard deviation of $S_{1}$, $S_{2}$, CCR, AA, GM and F-score metrics considering the variables of past criminal behaviors, sanctions, environment and situation of crime, and characteristics of crime.

	$S_{1}$	$S_{2}$	CCR	F-score	AA	GM
BayesNet	$77.10_{4.53}$	$\mathbf {82.36}_{\mathbf {5.21}}$	$78.83_{2.86}$	$82.97_{2.67}$	$79.73_{4.87}$	$44.90_{0.02}$
NaivesBayes	$75.31_{9.11}$	$78.77_{11.14}$	$76.45_{4.47}$	$80.84_{5.11}$	$77.04_{10.13}$	$41.95_{0.07}$
Logistic	$80.44_{3.92}$	$76.64_{6.14}$	$79.19_{2.78}$	$83.82_{2.20}$	$78.54_{5.03}$	$43.59_{0.02}$
SimpleLogistic	$79.93_{4.50}$	$\textit{80.30}_{\textit{5.08}}$	$80.05_{2.78}$	$84.28_{2.52}$	$80.11_{4.79}$	$45.38_{0.02}$
MultilayerPerceptron	$\textit{86.50}_{\textit{3.07}}$	$72.13_{5.69}$	$81.79_{2.33}$	$\textit{86.44}_{\textit{1.80}}$	$79.32_{4.38}$	$44.12_{0.01}$
RBFClassifier	$83.38_{3.66}$	$78.77_{5.44}$	$\textit{81.87}_{\textit{2.64}}$	$86.04_{2.18}$	$\textit{81.07}_{\textit{4.55}}$	$\mathbf {46.44}_{\mathbf {0.01}}$
SMO	$80.85_{4.61}$	$79.93_{5.19}$	$80.55_{3.03}$	$84.75_{2.66}$	$80.39_{4.90}$	$45.70_{0.02}$
IBk	$79.13_{3.25}$	$77.19_{5.86}$	$78.49_{2.57}$	$83.15_{2.10}$	$78.16_{4.56}$	$43.19_{0.01}$
KStar	$77.76_{4.21}$	$70.81_{6.21}$	$75.47_{3.29}$	$80.95_{2.78}$	$74.29_{5.21}$	$38.94_{0.02}$
LMT	$77.91_{4.46}$	$73.11_{8.46}$	$76.33_{3.37}$	$81.52_{2.78}$	$75.51_{6.46}$	$40.28_{0.03}$
RandomTree	$81.62_{3.71}$	$63.99_{8.46}$	$75.84_{3.00}$	$81.93_{2.24}$	$72.81_{6.09}$	$36.93_{0.02}$
RandomForest	$\mathbf {87.04}_{\mathbf {3.42}}$	$75.24_{5.73}$	$\mathbf {83.16}_{\mathbf {3.23}}$	$\mathbf {87.40}_{\mathbf {2.50}}$	$\mathbf {81.14}_{\mathbf {4.58}}$	$\textit{46.31}_{\textit{0.01}}$
J48	$72.62_{5.94}$	$76.78_{6.75}$	$73.98_{4.03}$	$78.83_{3.79}$	$74.70_{6.35}$	$39.42_{0.03}$
JRip	$77.55_{6.31}$	$74.89_{7.79}$	$76.67_{3.70}$	$81.59_{3.65}$	$76.22_{7.05}$	$41.07_{0.03}$

Significant values are in bold and italics.

## Discussion

The present study is pioneering in demonstrating the potential of Artificial Intelligence (AI) in extracting information from court decisions and accurately predicting Intimate Partner Femicide (IPF). From a data-focused standpoint, this study perceives legal text as a valuable source of information that can be assessed and analyzed using AI methods such as Natural Language Processing (NLP) and Machine Learning (ML) algorithms. Leveraging these techniques, patterns and connections within legal texts were identified, facilitating the prediction of IPF. This novel application of AI in the criminological field specifically focuses on utilizing legal texts within the domain of law.

The findings of this study suggest that the information extracted from court decision texts proves valuable in identifying risk indicators for IPF. The different algorithms employed in the study exhibited competitive performance in distinguishing between lethal and non-lethal violence in male-to-female intimate partner relationships. Specifically, it was observed that high accuracy in differentiating these groups can be achieved by considering a comprehensive set of variables, including criminal history, prior sanctions, characteristics of violence against women perpetrated by their intimate partners, as well as the environmental and situational context in which this violence occurs. These results align with existing literature emphasizing the significance of considering a combination of variables for effective crime detection^54–59^.

According with the literature, past criminal behavior is considered a strong predictor of future criminal behavior^29, 30^. Court judgments provides documented history of individuals’ involvement in criminal activities as well as their sanctions, and this information provides insights into recidivism. A first analysis was conducted with AI analyzing data of criminal records and sentences imposed on repeat offenders of violence against women in intimate partner relationships, and it detected patterns of repeat criminal behaviors related to IPF and the effectiveness of sentencing strategies on this behavior. However, this information was only able to detect about half of the cases of lethal and non-lethal violence suffered by women in intimate partner relationships. It indicates that criminal records and sanctions are relevant information to detect both types of violence, but there are cases where it is not sufficient to identify them. Some studies reveal that criminal records are a risk factor for IPF^31–33^, but no previous studies on the effect of sanctions on IPF have been identified, and the current study contributes in this regard. Our study reveals that the imposition of prior non-custodial sanctions is more related to LV than N-LV. In particular, the sanction of prohibition to approximate the victim is the one most associated with LV.

The aforementioned factors motivated the development of a second analysis, which incorporated environment and situational information pertaining to the occurrence of crimes against women. This expanded analysis enabled the detection of a higher number of cases involving both lethal and non-lethal violence. Analyzing crime incidents targeting women by their intimate partners across time and space yields crucial insights into the modus operandi and behavior of perpetrators involved in both types of violence. Specific geographic and temporal characteristics are associated with these criminal activities, distinguishing between lethal and non-lethal violence against women. Thus, IPF is influenced by context characteristics, and not only by individual factors of aggressors and victims^34, 35^. Previous research consistently demonstrates that the geographic location matters in the perpetration of IPF. Studies found higher rates of IPF in rural areas compared to urban areas^36–38^. This geographical disparity may be attributed to factors such as limited availability of resources, and reduced seeking help or intervention in situations of intimate partner violence^60–62^.

Additionally, prior research revealed that characteristics of the neighborhood also relates to IPF. For instance, limited community cohesion increases the risk of crime. By contrast, supportive social networks and strong social ties can act as protective factors in crime, including IPF^39, 40^. This is consistent with the findings of the current study. It found that people in the offender’s and victim’s immediate context have an influence on crime. Additionally, the study revealed that not only place and situational characteristics but also specific time periods play a crucial role in Intimate Partner Femicide (IPF) perpetration. The study identified distinct temporal patterns that differentiate IPF from non-lethal violence. Recognizing these temporal and geographical patterns is essential for implementing targeted interventions in specific areas and moments. This information facilitates the strategic allocation of resources, focusing not only on individuals at risk of experiencing IPF but also on specific locations and time periods associated with IPF.

The third analysis, in addition to incorporating the variables from the first and second analyses, included specific variables related to the characteristics of violence against women by their intimate partners. This comprehensive approach facilitated the detection of over three-quarters of both lethal and non-lethal violence cases. By considering factors such as the severity level, frequency, escalation, type (physical and/or psychological) of violence experienced by victims, and their coping strategies to address violence. This third analysis accurately identified a significant majority of both lethal and non-lethal violence cases. According to the scientific literature, in most cases, IPF is not an isolated event but rather the culmination of a pattern of escalating violence of abuse^41, 63, 64^. This could explain why variables related to severity, frequency, and escalation of violence lead to the detection of a greater number of IPF cases. Although physical violence has received the most attention in terms of predicting IPF^42, 63^, the present study, as well as a few others^33, 43^, demonstrates that psychological violence is also a significant determinant.

An additional local sensitive analysis of the RBFClassifier was conducted in order to further deeper insights into the specific variables among all ones analyzed that contribute to the correct classification of LV to N-LV^65^. The RBFClassifier was selected for this analysis because it is the algorithm that shows the most competitive performance on a single model, in contrast with the RandomForest, which is competitive but is an ensemble of models. The findings revealed that the discriminative power between both classes emerged when considering all variables collectively. Past criminal behaviors and sanctions imposed for their commission factors alone are insufficient to correctly classify LV and N-LV, as information is required on how violence against women occurs and the environment and situation in which it occurs. It has been identified that a specific criminal history of intimate partner violence (primarily criminal history of injuries, constraints, and habitual violence) is related to LV when physical and psychological violence is present, it becomes more frequent and severe, there were disputes prior to violence, and violence was exercised in places where there is commonly no nobody and social control is absent. In these cases, most of the victims have adopted disengagement coping strategies. This highlights the need to consider the overall characteristics to correctly identify LV and N-LV and not to draw general conclusions from a couple of factors.

The study not only offers valuable insights into IPF risk indicators but also extends the application of this knowledge to law enforcement professionals and related stakeholders, such as police and victim support services, who have access to the analyzed data. These professionals play a crucial role in identifying risk signals given the vast amount of data they handle, and our findings could assist them in this task. Consequently, these professionals would be able to promptly screen cases at risk of IPF, based on the validated risk indicators from our study, as soon as they obtain the case information. This information provides them with a foundation for urgent interventions by referring the cases to specialized services, implementing enhanced safety measures, and connecting victims with specialized support organizations, among other preventive actions.

The study has certain limitations that should be taken into account when interpreting the results. Firstly, the findings are based on specific Spanish legal documents, potentially limiting the generalizability of the findings to different legal contexts or document types. Secondly, the absence of long-term follow-up for the analyzed cases means that it is unknown whether instances of nonlethal violence identified during the study may escalate to lethal violence in the future. The study’s third limitation is the lack of qualitative information. Further longitudinal studies will be necessary to address this limitation and complement the preliminary findings of these analyses. It is important to note that the nature of the study made it impossible to extract information from interviews or other qualitative sources involving women who have experienced lethal violence. Therefore, the study relied solely on the analysis of available legal documents.

## Conclusions

This study pioneers the use of Artificial Intelligence (AI) to highlight the critical information present in legal documents for crime prediction, specifically focusing on male-to-female intimate partner violence. However, the approach employed has the potential to be extended to other types of crimes, marking new research inroads within the field of criminology. The study utilizes Natural Language Processing (NLP), a sub-field of AI, to extract court judgments related to male-to-female intimate partner violence from the Vlex legal database. Additionally, Machine Learning (ML), another branch of AI, is employed to identify specific elements in legal texts associated with Intimate Partner Femicide (IPF), which refers to lethal violence against women by male intimate partners.

The findings demonstrate that information regarding past criminal behaviors, imposed sanctions, the severity and frequency of violence against women, as well as the environmental and situational factors, successfully distinguish over three-quarters of both lethal and non-lethal violence within male-to-female intimate partner relationships. Integrating these findings into professional practices holds the potential to enhance the detection and prevention of IPF cases. Consequently, this study makes a valuable contribution to the field of criminology by expanding knowledge in the area of IPF and supporting effective prevention efforts.
